# Structural and Vibrational Characterizations of Alizarin Red S

**DOI:** 10.3390/molecules30153286

**Published:** 2025-08-05

**Authors:** César A. N. Catalán, Licínia L. G. Justino, Rui Fausto, Gulce O. Ildiz, Silvia Antonia Brandán

**Affiliations:** 1 Cátedra de Química General, Instituto de Química Inorgánica, Facultad de Bioquímica, Química y Farmacia, Universidad Nacional de Tucumán, Ayacucho 471, San Miguel de Tucumán 4000, Argentina; 2 CQC-IMS, Department of Chemistry, University of Coimbra, Rua Larga, 3004-535 Coimbra, Portugal; rfausto@ci.uc.pt; 3 Spectroscopy@IKU, Faculty of Sciences and Letters, Department of Physics, Istanbul Kultur University, Ataköy Campus, Bakirköy 34156, Istanbul, Turkey

**Keywords:** alizarin red S, molecular and electronic structure, harmonic force fields, vibrational analysis, DFT calculations

## Abstract

In this work, the structures of the isolated anion and anhydrous and monohydrated sodium salts of alizarin red S (ARS) have been theoretically investigated within the density functional theory framework (B3LYP/6-311++G** calculations). The combination of calculations with the scaled quantum mechanics force field (SQMFF) methodology has allowed the assignment of the experimental infrared spectrum of ARS in the solid phase and the determination of the corresponding force constants. The structural analysis also included the investigation of the NMR and UV-visible spectra of the compound in solution in light of the undertaken quantum chemical calculations, the obtained theoretical data being in good agreement with the corresponding experimental ones. The impact of the presence of the Na^+^ counterion and hydration water on the properties of the organic ARS^−^ fragment was evaluated. Atoms in molecules theory (AIM) analysis was also undertaken to obtain further details on the electronic structure of the investigated species, and the HOMO-LUMO gap was determined to evaluate their relative reactivity. Globally, the results obtained in this work extend the available information on alizarin red S and may also be used for the fast identification of the three studied species of the compound investigated (anhydrous and monohydrated sodium salts and isolated anion).

## 1. Introduction

**Figure 1:**
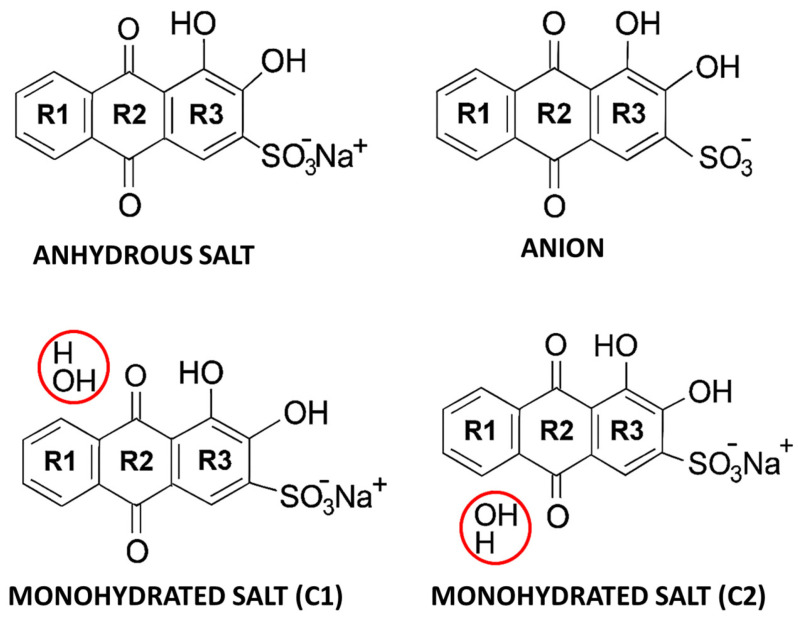
Structures of anion and anhydrous and monohydrated sodium salt of ARS with the adopted designation of the rings. Water molecules are highlighted by red circles.

Due to their chemical versatility and diversity of physical properties, anthraquinone derivatives have been receiving many different applications and have been investigated extensively [1,2,3,4,5,6,7,8,9,10,11,12,13,14,15,16,17,18,19,20,21,22,23,24,25,26]. Alizarin red S (ARS) is the water-soluble sodium salt of alizarin sulfonic acid (Figure 1), which was first synthesized by Graebe and Libermann in 1871 [1,2] and has been used in several domains mostly because of its ability to bind calcium ions and form characteristic colored red to orange complexes. Medical applications of ARS include its use as a probe for calcium deposits in tissues and the vascular system, for evaluation of bone growth, osteoporosis, or bone marrow characterization, in the study of cell signaling, gene expression, mesenchymal stem cells, etc. [3,4,5,6,7,8]. In geology, it is used to identify carbonate minerals, which stain at different rates [3,9]. In biology, to evaluate growth layers of living corals [10]. In industry, ARS receives applications as a dye for fabrics and in sensor manufacturing [11,12,13]. In chemistry and materials sciences, in the production of nanoparticles and metal complexes exhibiting properties suitable for different applications [14,15,16,17,18].

ARS has been extensively studied in relation to its function as a dye and also regarding its analytical determination, since its intensive use as a colorant has been raising some environmental concern [1,2,3,4,5,6,7,8,9,10,11,12,13,14,15,16,17,18,19,20,21,22,23,24,25,26]. On the other hand, the compound has been paid considerably less attention regarding its structural and vibrational properties. The experimental lattice parameters, space group, and crystal system of ARS were obtained by El-Nahass and co-workers [21] using powder X-ray diffraction (monoclinic, *Pc*, a = 15.69 Å, b = 7.83 Å, c = 14.07 Å, β = 113.39°), but, to the best of our knowledge, the crystal structure of the compound has not yet been solved, although it is known that in the solid state the compound tends to appear as a monohydrate, such as other dyes such as vialuric acid [27,28].

Theoretically, different tautomers of the ARS anion and their conformers have been recently investigated by Justino, Braz, and Ramos [18], using different levels of theory, in a study focusing on the Ga(III) complexes of the compound in semi-aqueous solution. Micro-Raman spectroscopy (MRS) and surface-enhanced Raman scattering (SERS) were used by Van Elslande, Lecomte, and Le Ho [26] to study several organic archaeologic colorants, including alizarin. The results evidenced that alizarin dyes are often difficult to analyze by conventional Raman spectroscopy due to extensive fluorescence interference, while, on the contrary, SERS appears as a convenient technique to successfully investigate this type of material [26]. The good performance of SERS to study alizarin dyes was also noted by Legan, Retkjo, and Ropret [1]. These last authors have investigated the degradation of ARS by both SERS and Fourier transform infrared (FTIR) spectroscopy [1] and found that degradation affects the hydroxyl, sulfonate, and carbonyl vibrations of the compound, though the obtained results could not be used for a quantitative analysis or extract detailed identification about the decomposition products [1]. In the above-mentioned study of El-Nahass and co-workers [21], a few bands observed in the FTIR spectra of microcrystalline ARS powder and amorphous thin films of the compound have been tentatively assigned. Nevertheless, a comprehensive study of the vibrational spectra of crystalline ARS, as well as of its isolated anion, has not yet been reported.

In the present study, infrared spectroscopy is used, together with B3LYP/6-311++G** calculations [29,30] and normal coordinate analysis, to perform the vibrational characterization of the anhydrous and monohydrated salts of alizarin red S (ARS-Na and ARS^-^Na/H_2_O) and of its anion (ARS^−^). Two possible structures have been considered for the monohydrated species, according to the structure of a similar disulfonate published by Jia [31] and also taking into account the known structure of monohydrated vialuric acid [27], as observed in Figure S1 (Supplementary Materials). Figure 1 shows the structures of the studied species of alizarin red S.

The most stable structures of these three species have been optimized in both the gas phase and those of ARS^−^ also in aqueous solution, and their structural (both geometric and electronic) properties evaluated. The detailed vibrational characterization of the different species has been performed, including the determination of their scaled harmonic force constants by using the transferable scaling factors approach [32,33,34]. Extensive comparison of the calculated data with experimental results has been completed in order to validate the calculations. Data include ^1^H and ^13^C NMR, infrared, and UV-visible spectra. Globally, the results obtained in this work extend the available information of alizarin red S and may also be used for the fast identification of the three studied species of the compound investigated (anhydrous and monohydrated sodium salts and isolated anion).

## 2. Materials and Methods

### 2.1. Experimental

Alizarin red S was purchased from Sigma-Aldrich and used as received. The experimental room temperature Attenuated Total Reflectance FTIR (ATR-FTIR) spectrum of the net polycrystalline compound was recorded in the 4000-400 cm*^−^*^1^ region using an iD7 ATR accessory in a Thermoscientific FTIR Spectrometer Nicolet iS5 (Thermo Fisher Scientific, Waltham, MA, USA), with a resolution of 2 cm*^−^*^1^ and 128 scans. The UV-visible absorption spectrum was obtained for the compound in aqueous solution (4.91 × 10*^−^*^5^ M; pH 4.1) using a Shimadzu UV-2100 spectrometer (Kyoto, Japan). The scanned wavelength range was from 200 to 700 nm with a step size of 1 nm. The experimental ^1^H and ^13^C NMR spectra of ARS were taken from Ref [18].

### 2.2. Computational Details

The initial conformations of the free ARS anion (ARS^−^) and of the anhydrous and monohydrates Na^+^ salts of the compound (ARS-Na and ARS-Na/H_2_O, respectively) have been modelled with the GaussView 6.1.1 program [35], taking into account the most stable conformer of the anion reported by Justino et al. [18]. These starting structures were then optimized in both the gas phase and also in water solution (for ARS^−^ and ARS-Na) using the Gaussian 16 program [36]. The hybrid B3LYP functional was used together with the 6-311++G** basis set [29,30], the calculations performed in water solution (including the calculation of the solvation energies) being performed with the integral equation formalism variant—polarizable continuum model (IEF-PCM) and the solvent model based on density (SMD) [37,38,39]. The B3LYP functional was chosen for the calculations considering that the main properties investigated in this work are geometries, charge distributions, and vibrational features, which are known to be reliably reproduced by B3LYP for the type of systems under consideration. Moreover, as it will be shown in the next sections of this article, the trends observed across the neutral, anionic, and salt forms are consistent and chemically meaningful. The B3LYP functional has been extensively and successfully used for similar systems in the literature, including neutral molecules, anions, and metal-organic salts, thus allowing a direct comparison of our results with a broader body of established work. Long-range corrected functionals are often preferred in studies involving charge-transfer states or systems with pronounced electron delocalization, but in the present study our focus is on ground-state structures and vibrational characteristics, where B3LYP has extensively demonstrated good performance. We shall also note that while the IEF-PCM/SMD continuum model provides a reliable approximation of bulk solvation effects, it does not explicitly account for specific solute-solvent interactions (such as hydrogen bonding). A more refined approach incorporating explicit solvent molecules may offer deeper insights into such interactions and can be considered in future work.

**Figure 2:**
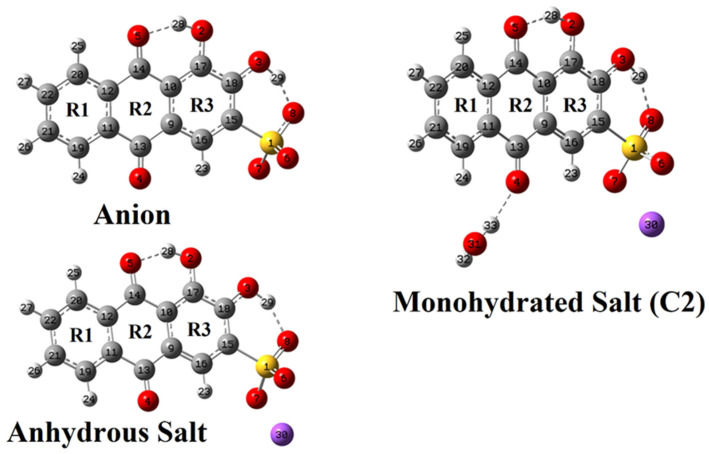
B3LYP/6-311++G** optimized structures of the free anion and anhydrous and monohydrated (most stable C2 form) salts of ARS with the used atom numbering scheme and naming of rings. Intramolecular H-bond interactions are shown as dashed lines.

Figure 2 shows the optimized structures of the anion and anhydrous and monohydrated salts of ARS calculated in the gas phase.

The Moldraw program was used to calculate the molecular volumes in the different media [40]. Natural atomic charges as well as the Merz-Kollman (MK) and natural population atomic (NPA) charges [41,42] were calculated with the NBO 5.1 program, while the molecules electron density topological properties were evaluated with the AIM 2000 program [43,44]. The studied systems were also investigated employing the frontier orbitals and a series of descriptors related to the chemical potential, electronegativity, global hardness, global softness, and global polarizability index [45,46].

The scaled quantum mechanical force field (SQMFF) methodology was used to obtain the harmonic force fields in order to perform the vibrational assignments of the different species at the B3LYP/6-311++G** level of theory [32,33,34] and following the procedure described elsewhere for similar compounds [47,48,49]. In the presented description of the modes, only potential energy distribution (PED) contributions *≥* 10% were considered. The vibrational assignments for the monohydrated species of ARS were also supported by comparing the obtained results with those available for compounds containing similar groups.

The B3LYP/6-311++G** ^1^H and ^13^C NMR spectra of the free anion in aqueous solution were calculated with the gauge-included atomic orbitals (GIAO) method [50], while the electronic absorbance spectrum was computed within the time-dependent DFT framework [28,51] and compared with the experimental one [36].

## 3. Results and Discussion

### 3.1. Calculated Total Energies, Dipole Moments, and Molecular Volumes of the Studied ARS Species in Different Media

Table 1 shows the B3LYP/6-311++G** calculated total zero-point vibrational energies (ZPVE), corrected energies, dipole moments, and molecular volumes of the three studied species in the gas phase as well as those of the ARS anion in aqueous solution. The optimized structures are shown in Figure 2. The calculations revealed that for ARS-Na/H_2_O, the most stable form corresponds to C2, so that only this form of the monohydrated salt has been considered further on.

As could be anticipated, Table 1 shows that the ARS anion has large dipole moment values in both media (gas phase and water solution), with a higher value in water because this charged species is highly hydrated in solution. The higher dipole moment in solution reflects the polarizing effect of the continuum solvent model but does not account for specific solute–solvent interactions such as hydrogen bonding, which are not being considered by the used theoretical model. In both monohydrated and anhydrous salts, the presence of Na^+^ leads to a reduction in the dipole moment, as compared with that of the anion, while, as it could be anticipated due to the increase of the number of atoms, it increases the molecular volume. Figure S2 (Supplementary Materials) shows the magnitude and direction of the dipole moment vectors for the three studied species in the gas phase. In all cases, the dipole moment vector stays in the main molecular plane, but the orientations are different. In the anion, the dipole moment is oriented nearly along the main molecular axis, pointing from the ring R3, which is heavily substituted with electronegative atoms containing groups, to R1 (following approximately the C12=C20 bond direction). On the other hand, in both the anhydrous and monohydrated salts, the dipole moment is oriented nearly perpendicular to the main molecular axis, crossing the R3 ring, mostly due to the presence of the positively charged Na^+^ ion. In the first case, it approximately bisects the C9=C16 bond, nearly pointing in the direction of O4, while in the monohydrated salt, the dipole moment vector is practically coincident with the C9=C10 bond and approximately bisects the O4**^…^**H23 distance. In water solution, the orientation of the dipole moment of the anion is identical to that for the gas phase (but it is larger in magnitude, as mentioned above).

### 3.2. ARS—Solvation Energy and Polarizability Change in Aqueous Solution

Calculated uncorrected (ΔG_un_) and corrected (ΔG_c_) solvation energies and polarizability change (Δα) relative to the gas phase of the ARS anion in aqueous solution are provided in Table 2. ΔG_un_ is calculated from zero-point corrected total energies obtained in solution and gas phase, while the corrected solvation energies include the contributions from the ΔG_ne_ term, accounting for the non-electrostatic interactions in solution. The solvation energy of the ARS anion in water has been calculated as −274.95 kJ mol^−1^, which is much larger than the calculated one for the hypothetical ARS-Na species (−155.95 kJ mol^−1^), thus confirming that the ARS-Na entity cannot subsist in solution, and only the dissociated hydrated ARS^−^ and Na^+^ ions exist. On the other hand, the calculated changes in the polarizabilities of the ARS^−^ and putative ARS-Na species in going from gas phase to aqueous solution are similar (see Table 2), a result that is in agreement with the very small polarizability of Na^+^ and its insensitivity to the media (the bare Na^+^ cation has a high and “hard” charge density, with its positive charge being concentrated within a small ionic radius of only ~0.95 Å in the gas phase and ca. 1.02 Å in water solution; 2.4–2.5 Å counting with the first hydration shell).

### 3.3. Geometries

Though the lattice parameters have already been reported for crystalline ARS by El-Nahass et al. [21], its detailed structure could not yet be solved. On the other hand, interpretation of vibrational spectra of crystalline ARS would be facilitated by the availability of structural data, in particular when, in the absence of detailed knowledge on the structure of the crystal, a simple approach based on calculated data for isolated species has to be used. Following these lines of reasoning, and in order to evaluate the appropriateness of the computed geometries for the vibrational calculations performed to support the vibrational spectra analysis, the calculated geometries for the ARS anion and the ARS-Na and ARS-Na/H_2_O entities were compared with available experimental crystal phase data for a compound structurally similar to ARS, specifically bis(1,10-phenanthrolin-1-ium) 9,10-di-oxo-9,10-dihydroanthracene-1,5-disulfonate hexahydrate [31] (Table 3). A graphical representation of the experimentally determined structure of this compound can be seen in Figure S1 (Supplementary Materials).

As seen in Table 3, the calculated geometries for ARS are in good agreement with the experimental reference data, with root mean square deviations (RMSD) in the ranges of 0.064-0.070 Å and 8.4-9.8^0^ for bond lengths and angles, respectively, the better values corresponding to the ARS-Na/H_2_O structure. In spite of the simplifications of the model and the fact that the experimental data refer to a different (though expectably structurally similar) molecule, these results indicate the relevance of the influence of both the Na^+^ ion and of the H_2_O molecule on the geometrical parameters of the ARS^−^ unit in the crystal. The calculations also demonstrate that the calculated geometries can be safely used in the vibrational calculations described in detail below.

### 3.4. ^1^H and ^13^C NMR Spectra

^1^H and ^13^C NMR spectra of ARS in solution (in 50% D_2_O/50% CD_3_OD and in CD_3_CN) have been reported before [18,52]. The GIAO/B3LYP/6-311++G** calculated data obtained in the present investigation are compared with the experimental data in Table 4 and Table 5. The experimental ^1^H-NMR spectrum of the compound in solution (50% D_2_O/50% CD_3_OD) recorded by Justino et al. [18] differs slightly from that reported by Sharma et al. [52] in CD_3_CN. In the first study, the OH protons could not be observed experimentally due to exchange with the solvent, while they give rise to singlet signals in the spectrum obtained in CD_3_CN solution [52]. The present calculations globally confirm the assignments performed in the experimental studies, though the assignment of H28 and H29 in [52] should be reversed. As seen in Table 4, with the noted exception, the calculated proton chemical shifts are in good agreement with the experimentally observed values, with an RMSD value of 0.33 ppm. The experimental ^13^C NMR spectrum is also fairly well reproduced by the calculations, with an RMSD value of 7.38 ppm. These RMSD values correspond to 3.5% and 5.0% average errors for the ^1^H and ^13^C calculated chemical shifts, respectively. In relation to the predicted ^13^C chemical shifts, it can be seen in Table 5 that the larger deviations occur for the carbon atoms directly connected to O and S atoms. The less good agreement between the calculated and experimental values in these cases is likely due to a combination of strong electronic effects exerted by the electronegative atoms and limitations in the used computational method to account for electron delocalization (B3LYP tends to over delocalize electron density, especially in π-systems or near lone pairs, with O and S being particularly problematic due to the usual involvement of lone pairs in conjugation), and in particular hydrogen bonding.

### 3.5. Atomic Charges and Bond Orders

Atomic Merz-Kollman (MK) and natural population atomic (NPA) charges and bond orders (BO) [41,42,43,44] were calculated for the relevant ARS species. These results are summarized in Table 6, highlighting the data for the heteroatoms and the H atoms of the OH groups, which are involved in H-bonding interactions. In Figure S3 (Supplementary Materials), MK and NPA data are also provided in graphical format.

The calculations show that in the gas phase the charges on the sulfonate oxygen atoms increase in absolute value (become more negative) when in the presence of the Na^+^ ion, as expected, while that of the sulfur atom tends to become less positive (Table 6). These trends are observed in both calculated MK and NPA charges, which globally agree almost quantitatively with each other, though for the sulfonate group the MK charges predict the charge on the sulfur atom to be less positive and those of the oxygen atoms to be less negative for all studied systems (see Figure S3, Supplementary Materials). This latter behavior is also observed for the calculated charges of the ARS anion in aqueous solution. In this species, the calculated charges tend to increase in absolute value, in agreement with the calculated larger dipole moment for ARS^−^ in aqueous solution compared to the gas phase. It shall be noticed that both charge partition methods assign the sulfur atom a large positive charge, due to the fact that this atom is bound to 3 oxygen atoms and accounting for the large polarizability of the sulfur. Very interestingly, this is also observed for both Na^+^ containing species in spite of the reduction in the charge of the sulfur atom in these cases, as noticed above.

The calculation of the sum of bond orders per atom (BO), expressed as the Wiberg bond index (Table 6), reveals that this parameter slightly reduces in both the sulfur and sulfonate oxygen atoms in the presence of the Na^+^ cation, while slightly increasing in the remaining oxygen atoms. For the ARS anion, the values in the gas phase and in aqueous solution do not differ significantly. It is interesting to note that O8, which stays close to the hydroxy H29 atom, has a significantly lower BO (1.530 and 1.515 in the gas phase and in aqueous solution, respectively) than both O6 and O7 in the anion (1.603/1.594 in the gas phase and 1.587/1.579 in water solution), while the inverse order is observed for the salts. These results show that the intramolecular O3-H29···O8 H-bond existing in the ARS anion (which pulls electron charge from the S-O8 bond to the O8 atom) loses importance in the presence of the Na+ ion. In addition, the presence of the cation leads to a reduction of the BO in all sulfonate oxygen atoms by pulling electrons from the S-to-O bonds to the corresponding oxygen atoms, but this effect is attenuated in the case of the interaction with the O8 atom involved in the intramolecular H-bond due to competition with this latter interaction. These conclusions are in agreement with the H-bonding structural data for the anion and salts shown in Table 7.

### 3.6. AIM Analysis

**Figure 3:**
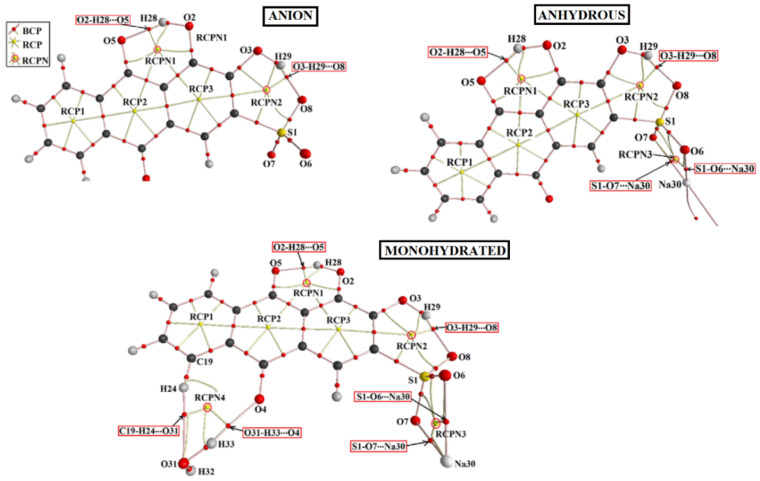
Details of the molecular graphs for the anion, anhydrous, and monohydrated salts of ARS in the gas phase, built based on the corresponding B3LYP/6-311++G** wavefunction and optimized geometries. Bond critical points (BCPs) and ring critical points (RCPs) are shown, together with the bond paths.

The topological properties of the electron density ρ(r) and the Laplacian of the electron density ∇^2^ρ(r) were investigated within the framework of the AIM theory. Relevant results for these functions at bond critical points (BCPs) and ring critical points (RCPs) [37,38] are provided in Table S1 (Supplementary Materials). Figure 3 shows the molecular graphs obtained for the anion and both salts of ARS in the gas phase. A similar graph was obtained for the anion in water solution, and, for this reason, it is not presented in the figure. RCP1, RCP2, and RCP3 are the RCPs of the R1, R2, and R3 rings, respectively, while RCPNs represent those RCPs associated with the pseudo-rings that include either intramolecular H-bonds or O-to-Na ionic interactions. The anhydrous salt presents two H bonds and two ionic interactions (S1-O6···Na and S1-O7···Na), while the monohydrated salt bears these four non-covalent interactions plus two additional ones due to H bonds with the water molecule. Three and four RCPNs are observed in the anhydrous and monohydrated salts, respectively, demonstrating that the AIM theory duly accounts for the pseudo-rings involving non-covalent interactions.

**Figure 4:**
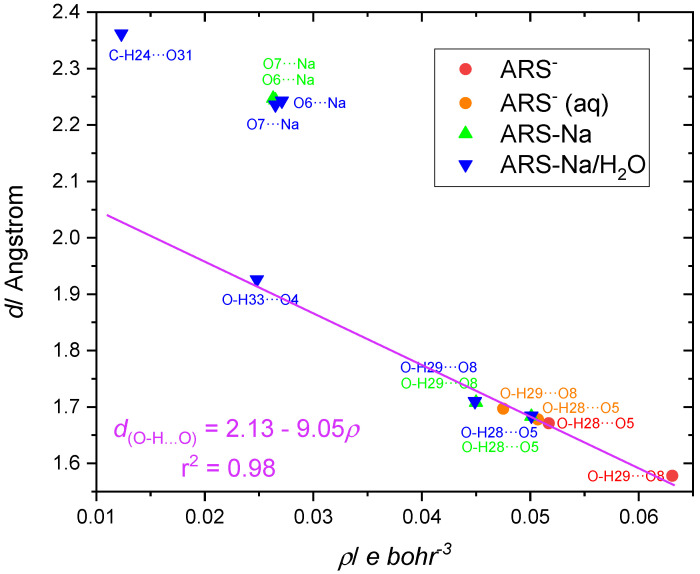
Plot of the electron density at the BCPs associated with the non-covalent interactions (H-bonds and ionic S-O···Na interactions) in the different species studied as a function of the corresponding distance.

The values of the electron density at the BCPs associated with the non-covalent interactions (H-bonds and ionic S1-O6···Na and S1-O7···Na interactions) are plotted as a function of the corresponding distance in Figure 4. The electron density values at the BCP are small (less than 0.07 *e* bohr^−3^) and ∇^2^ρ(r) are positive (i.e., there is a depletion of electron density between nuclei) (see Table S1, Supplementary Materials) in agreement with the non-covalent nature of all these interactions [37,38]. For the O-H···O bond interactions, a linear correlation was found with a high correlation coefficient (r^2^ = 0.98), with the shorter H-bond lengths having higher electron density at the BCP, as expected. The C-H···O interaction present in ARS-Na/H_2_O appears in the plot as a well-separated point with a longer distance and smaller electron density value at the associated BCP, also as expected. In turn, all S-O···Na interactions appear closely clustered in the graph, demonstrating that these interactions are not affected by the presence of the hydration water in ARS-Na/H_2_O.

Interestingly, for all RCPs and RCPNs observed in the different species, except that associated with the pseudo-ring involving the hydration water in ARS-Na/H_2_O, the electron density at the critical point is similar (between 0.22 and 0.17 *e* bohr^−3^), indicating that both the O-H···O hydrogen bonds and S-O···Na ionic interactions present in the pseudo-rings are substantially strong. The much smaller value for the electron density at the RCPN associated with the pseudo-ring involving the hydration water in ARS-Na/H_2_O (~0.008 *e* bohr^−3^; see Table S1, Supplementary Materials) demonstrates the fact that this pseudo-ring is comparatively labile, due to the weak C-H···O interaction.

### 3.7. Frontier Orbitals, HOMO-LUMO Gap, Electronic Reactivity Descriptors, and UV-vis Spectrum

The HOMO-LUMO energy gap is a good parameter to predict electronic reactivity. Assuming that the HOMO and LUMO energies represent the symmetrical of the ionization potential and electron affinity, respectively (Koopman’s Theorem), the HOMO-LUMO gap can be used to calculate different reactivity-related molecular descriptors such as the chemical potential (*μ* = [E_(LUMO)_ + E_(HOMO)_]/2), molecular electronegativity (*χ* = −[E_(LUMO)_ + E_(HOMO)_]/2), global hardness (*η* = [E_(LUMO)_ − E_(HOMO)_]/2), global softness [*S* = 1/(2 *η*)], and global polarizability index [ω = *μ*^2^/(2*η*)] descriptors [45,46]. Table 8 shows the calculated HOMO-LUMO gap and the corresponding calculated values for these descriptors.

**Figure 5:**
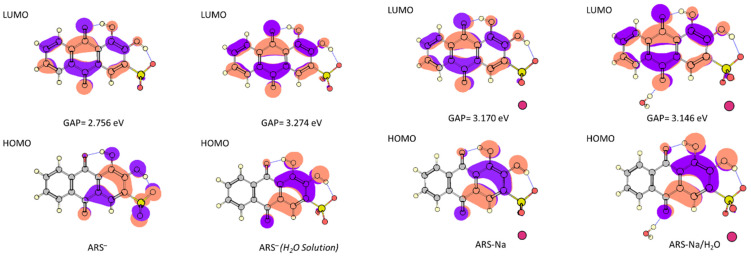
B3LYP/6-311++G** calculated HOMO and LUMO orbitals and HOMO-LUMO gap values for the most stable forms of the anion, anhydrous and monohydrated salts of ARS in the gas phase, and, for the anion, also in aqueous solution.

The calculated HOMO-LUMO gap values show that, in the gas phase, the anion is the most reactive (smaller energy gap value) species, while the salts are predicted to have similar reactivities. For the anion, when going from gas phase to water solution, the HOMO-LUMO gap increases, indicating that this species is comparatively more stable in aqueous solution, as could be expected. Figure 5 shows the HOMO and LUMO orbitals for the investigated systems in the gas phase, and for the anion, also in aqueous solution. The results for both salts show that the HOMO for these systems is mainly localized on R3, while the LUMO extends to all three rings, both orbitals being π-type orbitals. In the case of the anion, the frontier orbitals are identical to those of the remaining species when the anion is in aqueous solution, while its HOMO is also extended to the sulfonate group for the anion in the gas phase, implying that in this latter situation the LUMO ← HOMO transition should comprehend some degree of sulfonate-to-rings charge transfer. In all cases, some charge transfer character from R3 to R2/R1 can be expected for the LUMO ← HOMO transition.

**Figure 6:**
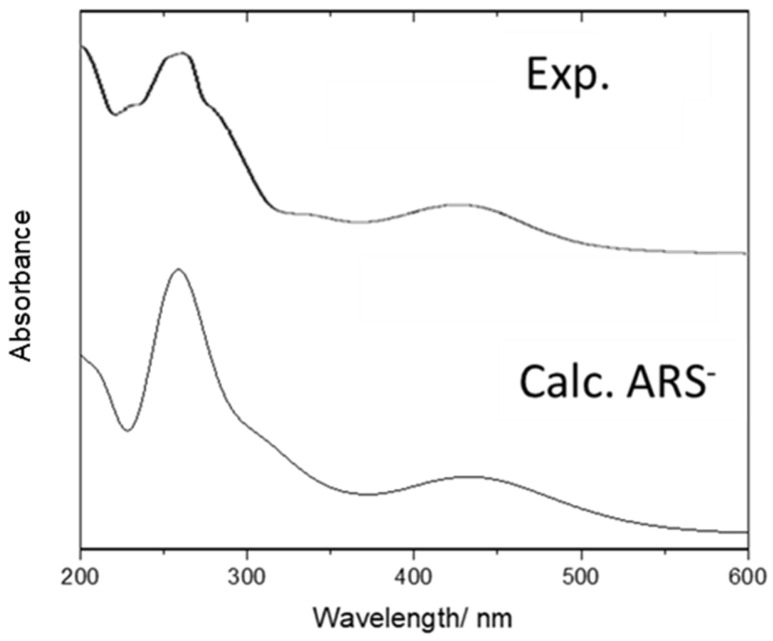
Experimental UV-visible spectrum of ARS in aqueous solution compared with the TD-DFT/B3LYP/6-311++G** predicted spectrum for ARS^−^ (in H_2_O).

The experimental electronic spectrum of ARS in aqueous solution, obtained for a concentration of 4.91 × 10^−5^ mol dm^−3^ and pH 4.1, is compared in Figure 6 with the TD-DFT/B3LYP/6-311++G** predicted spectra for the anion of the compound in aqueous solution. The experimental spectrum for ARS shows bands at 259 nm (with shoulders at 233 and 276 nm), 333 nm, and 423 nm, with the calculated spectrum of ARS^−^ reproducing the experimental one very well. Assignments are being presented in Table 9.

### 3.8. Vibrational Analyses

**Figure 7:**
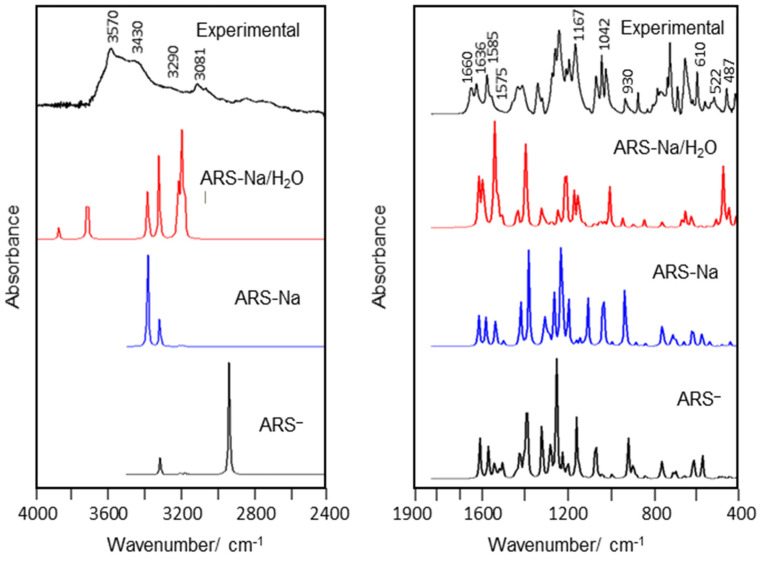
Solid state room temperature experimental ATR-IR spectrum of ARS (in the 4000–2400 and 2000–400 cm**^−^**^1^ regions, compared with the corresponding B3LYP/6-311++G** predicted infrared spectra for the isolated anion and anhydrous and monohydrated salts).

The lowest energy structures for the anion and anhydrous and monohydrated salts of ARS belong to the *C*_1_ symmetry. For the sulfonate group we used a local *C*_s_ symmetry. For the anhydrous and monohydrated salts, 84 and 93 normal vibration modes exist, respectively, while for the anion there are 81 modes, all active in infrared. Figure 7 shows comparisons of room temperature experimental (ATR-IR) spectra of ARS in the solid phase in different regions with the B3LYP/6-311++G** predicted spectra for the isolated ARS anion and anhydrous and monohydrated salts.

Though the calculations have been performed for the isolated species so that intermolecular interactions are not considered, the calculated spectrum for the monohydrated salt shows a fairly good correlation with the experimentally observed one, notably considerably better than those predicted for the isolated ARS ion and ARS-Na species, thus demonstrating the relevance of considering both the Na^+^ counterion and the hydration water in the simulations**.** We shall note that the spectral region below 400 cm^−1^ was not investigated, so that calculated modes appearing below this frequency value could not be validated.

A tentative vibrational assignment of the experimental bands has been performed using the SQMFF methodology and the potential energy distribution (PED) obtained using the Molvib program [32,33,34]. These results are summarized in Table 10, wherein the assignments only PED contributions ≥ 10% are indicated. Calculated data for the ARS^−^ and ARS-Na isolated species and full calculated wavenumbers for ARS-Na/H_2_O are provided in Table S2 (Supplementary Materials). Discussions on assignments of the most important modes are given below.

#### 3.8.1. Band Assignments

4000–2400 cm^−1^ region

The IR bands at 3570 and 3430 cm**^−^**^1^ are straightforwardly assigned to antisymmetric and symmetric O-H stretching modes of water, while the bands at 3290 and 3081 cm**^−^**^1^ are assigned to O3-H29 and O2-H28 stretching modes. The assignments of OH stretching modes of water are in agreement with those observed for similar hydrated salts [28,51]. Note that the calculations predict the O3-H29 and O2-H28 stretching modes at lower wavenumbers because these groups are involved in intramolecular H-bonds, as supported by the AIM calculations. The bands between 3081 and 2807 cm**^−^**^1^ are assigned to C-H stretching modes (and overtones and combination modes of the CH bending vibrations).

1700–1000 cm**^−^**^1^ region

The C13=O4 and C14=O5 stretching modes give rise to the strong IR bands at 1660 and 1636 cm**^−^**^1^, respectively, in good agreement with the SQM predicted values (1636 and 1623 cm^−1^, respectively). On the other hand, C=C stretching modes of the aromatic rings are predicted between 1575 and 1520 cm**^−^**^1^, and assigned to the strong experimental IR band at 1585 cm**^−^**^1^ and shoulder at ca. 1575 cm^−1^. The deformation O-H mode of water is predicted at 1546 cm**^−^**^1^ and considered to contribute also to the first of these two bands [28,51]. The SQM calculations predicted the O3-H29 deformation mode at 1421 cm**^−^**^1^, which can be made in correspondence with the shoulder observed at 1466 cm**^−^**^1^. The antisymmetric and symmetric stretching modes of the sulfonate group involving the two oxygen atoms bound to Na^+^ were predicted at 1121 and 944 cm**^−^**^1^, respectively, and assigned to the bands at 1167 and 930 cm**^−^**^1^, in good agreement with literature data [47,48,49]. On the other hand, the S1-O6 stretching mode is predicted at 1044 cm**^−^**^1^ and assigned to the intense IR band at 1042 cm**^−^**^1^.

1000–400 cm**^−^**^1^ region

The C-C stretching modes as well as the deformational modes of the sulfonate group and deformations and torsions of the three rings are expected to give rise to bands in this region [47]. Most of these modes are predicted to be extensively mixed modes. The modes predicted to have larger contributions of the deformational modes of the sulfonate group are predicted at 630 (wagg SO_2_), 527, and 478 (both modes being mixed twist/bend SO_2_ vibrations) cm**^−^**^1^, and were ascribed to the experimental bands observed at 610, 522, and 487 cm**^−^**^1^, respectively. The librations of the water molecule are predicted at low wavenumbers, as it is commonly observed in hydrated species [28,51]. The same applies to most of the deformational and torsional modes of the rings [28,47,48,49], with many of these vibrations being expected to appear below 400 cm^−1^, i.e., in a spectral region not investigated (see Table S2 in the Supplementary Materials).

#### 3.8.2. Force Fields

The scaled force constants are useful parameters to characterize the strengths of the different bonds. They were computed at the B3LYP/6-311++G** level of theory for the studied ARS species in the gas phase by using the SQMFF methodology and the Molvib program [32,33,34]. The results are shown in Table 11 and show clear differences between the salts and the isolated anion. For the salts, only minor differences are noticeable: in the *f*(νC=O) force constants, because the water molecule in the monohydrated salt is forming an H-bond with one of the C=O bonds (hence, the higher value for *f*(νC=O) observed for the anhydrous species), and in the *f*(δOH)_H-bonded_ force constant, which is also larger in the anhydrous species than in the monohydrated salt, for an identical reason. Note that the force constants related to the stretching modes of the sulfonate group are similar in both salts but different from the anion because of the presence of the Na^+^ counterion in the salts, which is also reflected in different local symmetries (*C*_s_ and *C*_3V_, respectively). The *f*(νS=O) force constant in the anion is slightly larger than those for both salts.

## 4. Conclusions

In this study, the structures of the isolated anion, as well as the anhydrous and monohydrated sodium salts of alizarin red S (ARS), have been theoretically investigated using density functional theory (DFT) calculations at the B3LYP/6-311++G** level. The calculated solvation energies confirmed that only hydrated ARS^−^ and Na^+^ ions should exist in water solution, while the calculated structure for the ARS-Na species was found to fit well the existing experimental one for a similar reference compound in crystalline phase (in the absence of crystal data for ARS). The combination of these calculations with the scaled quantum mechanics force field (SQMFF) methodology has enabled the assignment of the experimental infrared spectrum of ARS in the solid phase and the determination of the corresponding force constants.

In addition to structural analysis, the study also explores the NMR and UV-visible spectra of ARS in solution, leveraging quantum chemical calculations to interpret the results. The obtained theoretical spectral data show strong agreement with experimental findings, also reinforcing the validity of the used computational approach. Furthermore, the impact of the Na**^+^** counterion and hydration water on the electronic and structural properties of the ARS fragment was assessed through analysis of Merz-Kollman (MK) and natural population atomic (NPA) charges and bond orders (BO).

To gain deeper insights into the electronic structure of the studied species, an Atoms in Molecules (AIM) analysis was conducted, providing additional details on their bonding characteristics. Additionally, the HOMO-LUMO gap and related electronic reactivity parameters were determined to evaluate their relative reactivity.

Overall, the results of this work expand the existing knowledge of alizarin red S and offer valuable reference data for the rapid identification of the three investigated species—the isolated anion, anhydrous sodium salt, and monohydrated sodium salt.

## Figures and Tables

**Table 1:** B3LYP/6-311++G** calculated total and corrected by ZPVE energies (E and E_ZPVE_, respectively), dipole moments (µ), and molecular volumes (V) for the anion, anhydrous, and monohydrated sodium salts of ARS in the gas phase and for the anion in aqueous solution.

Species	E (Hartrees)	E_ZPVE_ (Hartrees)	µ (D)	V (Å^3^)
GAS PHASE				
ARS^−^	−1462.8628	−1462.6725	15.19	273.3
ARS-Na	−1625.1363	−1624.9439	9.16	314.5
ARS-Na/H_2_O	−1701.6045	−1701.3879	7.69	335.7
AQUEOUS SOLUTION				
ARS^−^	−1462.9580	−1462.7684	23.34	273.4

**Table 2:** B3LYP/6-311++G** calculated corrected solvation energies (ΔG_C_) and uncorrected by ZPVE energies (ΔG_un_) (in kJ mol^−1^) and polarizability variations relative to gas phase (Δα; a.u.) of the ARS anion and hypothetical ARS anhydrous Na^+^ salt (ARS-Na). Calculations were performed using the SMD solvation model.

Species	ΔG_un_	ΔG_ne_	ΔG_C_	Δα
ARS^−^	−251.54	23.41	−274.95	113.973
ARS-Na	−132.46	23.49	−155.95	119.769

**Table 3:** B3LYP/6-311++G** calculated geometrical parameters for the anion, anhydrous, and monohydrated sodium salts of ARS in the gas phase, compared with experimental data for bis(1,10-phenanthrolin-1-ium) 9,10-di-oxo-9,10-dihydroanthracene-1,5-disulfonate hexahydrate [31].

Theoretical ^a^				Exp. ^b^
Parameter	ARS^−^	ARS-Na	ARS-Na/H_2_O	
Bond Length (Å)				
S1-C15	1.831	1.801	1.801	1.800
S1-O6	1.474	1.503	1.503	1.444
S1-O7	1.472	1.501	1.500	1.444
S1-O8	1.509	1.479	1.479	1.438
C13-O4	1.226	1.224	1.230	
C14-O5	1.246	1.239	1.239	
C17-O2	1.337	1.331	1.330	
C18-O3	1.328	1.330	1.329	1.211
C15-C16	1.396	1.397	1.396	
C15-C18	1.401	1.400	1.400	1.409
C17-C18	1.428	1.432	1.432	1.486
C10-C17	1.408	1.404	1.404	1.493
**RMSD**	**0.069**	**0.070**	**0.064**	
Bond angle (°)				
C15-S1-C6	104.98	107.17	107.13	108.16
C15-S1-C7	105.68	107.11	107.22	103.58
C15-S1-C8	102.12	105.56	105.34	106.05
C16-C15-C18	120.80	121.35	121.39	
C16-C15-S1	118.45	117.52	117.89	
C18-C15-S1	120.67	121.10	120.66	126.18
C17-C18-C15	118.65	118.08	118.12	119.09
C15-C18-O3	124.18	125.74	125.61	121.98
C17-C18-O3	117.15	116.17	116.26	118.84
C10-C17-C18	120.37	120.23	120.15	119.09
C9-C13-O4	123.31	121.96	120.89	
C11-C13-O4	119.63	120.94	121.53	
C12-C14-C10	118.26	118.28	118.23	
C12-C14-O5	119.38	120.29	120.41	
C10-C14-O5	122.34	121.42	121.34	
C9-C13-C11	117.05	117.08	117.56	
**RMSD**	**9.8**	**8.4**	**8.4**	

^a^ This work. ^b^ Data for bis(1,10-phenanthrolin-1-ium) 9,10-di-oxo-9,10-dihydroanthracene-1,5-disulfonate hexahydrate) [31]. The RMSD values are indicated in bold letters.

**Table 4:** Comparison between observed [18,52] and B3LYP/6-311++G** calculated ^1^H chemical shifts (δ, in ppm) for ARS^−^ in aqueous solution.

Atom	Theoretical ^a^	Experimental ^b^	Deviation
H23	8.56	8.02	0.54
H24	8.42	8.32	0.10
H25	8.36	8.27	0.09
H26	7.47	7.90	−0.43
H27	7.44	7.90	−0.46
H28	12.82	12.80	0.02
H29	11.72	11.52	0.20
**RMSD**	**0.33**		

^a^ This work, GIAO/B3LYP/6-311++G**; Ref. to TMS, ^b^ from [18,52]; in [52], the assignments for H28 and H29 are the reverse ones. The RMSD values are indicated in bold letters.

**Table 5:** Comparison between observed [18,52] and B3LYP/6-311++G** calculated ^13^C chemical shifts (δ, in ppm) for ARS^−^ in aqueous solution.

Atom	Theoretical ^a^	Experimental ^b^	Deviation
C9	125.55	122.80	2.75
C10	118.70	116.53	2.17
C11	139.25	131.99	7.26
C12	139.01	132.60	6.41
C13	185.68	187.92	−2.24
C14	193.48	181.52	11.96
C15	148.24	133.36	14.88
C16	130.23	120.15	10.08
C17	159.97	150.91	9.06
C18	157.87	149.80	8.07
C19	131.83	127.00	4.83
C20	130.78	127.38	3.4
C21	136.13	135.47	0.66
C22	134.47	136.37	−1.9
**RMSD**	**7.38**		

^a^ This work, GIAO/B3LYP/6-311++G**; Ref. to TMS, ^b^ from [18,52]. The RMSD values are indicated in bold letters.

**Table 6:** B3LYP/6-311++G** calculated Merz-Kollman (MK) and natural population analysis (NPA) atomic charges (in units of electron; *e =* 1.602 × 10^−19^ C) on selected atoms and sum of bond orders per atom (BO; expressed as Wiberg indexes), for the relevant species of ARS in gas phase and aqueous solution.

ARS^−^							
Gas Phase				Aqueous Solution		
Atoms	MK	NPA	BO	MK	NPA	BO
1 S	1.311	2.299	4.317	1.340	2.288	4.319
2 O	−0.597	−0.653	2.016	−0.602	−0.655	2.008
3 O	−0.595	−0.665	2.007	−0.603	−0.668	1.992
4 O	−0.554	−0.576	2.008	−0.548	−0.572	2.006
5 O	−0.615	−0.639	1.980	−0.621	−0.636	1.983
6 O	−0.654	−0.955	1.603	−0.666	−0.961	1.587
7 O	−0.649	−0.959	1.594	−0.663	−0.965	1.579
8 O	−0.718	−1.015	1.530	−0.717	−1.021	1.515
28 H	0.460	0.497	0.759	0.468	0.498	0.758
29 H	0.482	0.510	0.746	0.471	0.515	0.740
**ARS-NA**				**ARS-NA/H_2_O**		
**Gas Phase**				**Gas phase**		
**Atoms**	**MK**	**NPA**	**BO**	**MK**	**NPA**	**BO**
1 S	1.183	2.285	4.291	1.108	2.304	4.300
2 O	−0.550	−0.638	2.032	−0.505	−0.634	2.035
3 O	−0.556	−0.642	2.027	−0.513	−0.636	2.030
4 O	−0.506	−0.570	2.012	−0.510	−0.617	1.981
5 O	−0.543	−0.607	2.009	−0.538	−0.607	2.006
6 O	−0.719	−1.038	1.475	−0.699	−1.039	1.478
7 O	−0.705	−1.038	1.474	−0.685	−1.039	1.475
8 O	−0.608	−0.963	1.596	−0.591	−0.965	1.594
28 H	0.446	0.503	0.753	0.433	0.495	0.762
29 H	0.483	0.512	0.743	0.470	0.506	0.750
30 Na	0.898	0.960	0.082	0.896	0.948	0.105
31 O				−0.747	−0.918	1.603
32 H				0.359	0.439	0.814
33 H				0.373	0.476	0.781

**Table 7:** B3LYP/6-311++G** calculated intramolecular H-bond distances (d/Å) and angles (α/^o^) in the ARS anion and anhydrous and monohydrated salts in the gas phase and of the ARS anion in aqueous solution by using the same level of theory.

Gas Phase				
Species	d(O2-H28···O5)	α(O2-H28···O5)	d(O3-H29···O8)	α(O3-H29···O8)
ARS^−^	1.671	148.2	1.578	156.4
ARS-Na	1.683	147.0	1.708	150.3
ARS-Na/H_2_O	1.684	146.9	1.710	150.2
Aqueous Solution				
ARS^−^	1.678	146.8	1.697	152.0

**Table 8:** B3LYP/6-311++G** calculated energies (eV) of the frontier molecular orbitals (HOMO and LUMO), HOMO-LUMO gap energies, and descriptors for the anion and anhydrous and monohydrated salts of ARS.

Orbital	ARS^−^		ARS-Na	ARS-Na/H_2_O
	Gas Phase	Aqueous Solution	Gas Phase	Gas Phase
HOMO	−3.5157	−6.5731	−6.3682	−6.3593
LUMO	−0.7592	−3.2988	−3.1980	−3.2137
│GAP│	2.7561	3.2743	3.1702	3.1456
*χ*	2.1375	4.9359	4.7831	4.7865
*μ*	−2.1375	−4.9359	−4.7831	−4.7865
*η*	1.3783	1.6371	1.5851	1.5728
*S*	0.3628	0.3054	0.3154	0.3179
*ω*	1.6574	7.4409)	7.2166	7.2834

**Table 9:** Positions of bands observed in the UV-visible spectrum of ARS in aqueous solution at 4.91 × 10^−5^ mol dm^−3^ and pH 4.1, and the TD-DFT/B3LYP/6-311++G** predicted spectrum of ARS^−^ in aqueous solution.

Exp./nm	Calculated/nm (ARS^−^)	*f*	Assignment	%
423 w	439.5	0.1749	S_0_ → S_1_ ππ* HOMO → LUMO	70
	393.9	0.0001	S_0_ → S_2_ ππ* HOMO-3 → LUMO	69
	378.4	0.0452	S_0_ → S_3_ ππ* HOMO-1 → LUMO	69
	350.6	0	S_0_ → S_4_ *n*π* HOMO-6 → LUMO	68
333 w	341.6	0.0789	S_0_ → S_5_ ππ* HOMO-2 → LUMO	69
	321.4	0.0009	S_0_ → S_6_ *n*π* HOMO-4 → LUMO	70
	306.5	0.0818	S_0_ → S_7_ ππ* HOMO → LUMO+1	69
276 sh	302.7	0.1804	S_0_ → S_8_ ππ* HOMO-5 → LUMO	68
	267.6	0.0017	S_0_ → S_9_ ππ* HOMO-3 → LUMO+1	66
259 vs	264	0.5645	S_0_ → S_10_ ππ* HOMO-1 → LUMO+1	63
	260.8	0.0004	S_0_ → S_11_ *n*π* HOMO-7 → LUMO	68
	250.7	0.0113	S_0_ → S_12_ ππ* HOMO-2 → LUMO+1	60
233 sh	249.7	0.3648	S_0_ → S_13_ ππ* HOMO-1 → LUMO+2	51

**Table 10:** Observed (room temperature solid ATR-IR spectrum of crystalline ARS monohydrate sodium salt) and calculated (SQMFF, B3LYP/6-311++G**) wavenumbers (cm**^−^**^1^) and assignments for the isolated ARS-Na/H_2_O species *^a^***.**

	Calculated			Calculated	
Exp.	ARS-Na/H_2_O	Assignment *^b^*	Exp.	ARS-Na/H_2_O	Assignment *^b^*
3570 s	3730	ν_a_OH(W)	1042 vs	1044	νC21-C22
3430 s	3519	ν_s_OH(W)	1020 s	1019	γC21-H26, γC19-H24
3290 m	3240	νO3-H29		1011	νC21-C22, νC20-C22
3081 m	3175	νO2-H28	987 sh	1000	γC20-H25, γC22-H27
	3082	νC16-H23	930 m	944	ν_s_SO_2_
	3072	νC20-H25	912 sh	913	γC20-H25, γC19-H24
	3069	νC19-H24		905	βC13-O4
3034 sh	3055	νC21-H26	899 sh	898	γC16-H23
2807 sh	3041	νC22-H27	868 s	854	νC17-C18
1660 s	1636	νC13=O4	799 sh	801	γC22-H27, γC21-H26
1636 s	1623	νC14=O5	778 s	771	βR_1_(A2)
1585 s	1573	νC9-C16	762 sh	769	τR_1_(A1), τR_1_(A2), γC13-O4
	1571	νC19-C21	731 sh	740	τR_2_(A3), γC17-O2, γC18-O3
1575 sh	1550	νC21-C22, νC14=O5		729	βR_2_(A1)
1585 s	1546	δOH(W)	717 vs	726	τR_1_(A3), γC18-O3
1466 sh	1471	δO3-H29, νC9-C10		705	τwO2-H28
	1461	δO3-H29, βC19-H24	682 s	682	τwO3-H29
	1460	βC16-H23		673	βR_3_(A1)
1440 s	1437	βC22-H27, βC21-H26		671	τR_1_(A1), τR_2_(A1)
1417 s	1421	δO3-H29, νC15-C18	645 vs	655	βR_2_(A3), βC18-O3
1347 s	1351	νC10-C14, νC10-C17, δO2-H28	610 w	630	wagSO_2_
1319 m	1327	νC17-O2, βR_1_(A3)	589 s	590	τR_3_(A3), γC15-S1
	1312	νC11-C19, νC12-C20, νC11-C12		587	τR_3_(A3), γC15-S1
1263 s	1296	νC9-C13	551 w	561	τH33-O4, τO31-H33
	1265	βC19-H24		550	τR_1_(A3)
1244 vs	1255	νC16-C15, νC18-O3	522 sh	527	τwSO_2_, δSO_2_
1208 s	1225	νC12-C14	508 m	510	βR_3_(A3), βR_2_(A1)
1196 s	1189	νC11-C13	487 sh	478	δSO_2_, τwSO_2_
1167 vs	1167	βC16-H23		472	βR_3_(A2), βR_3_(A3)
	1156	βC21-H26, βC22-H27, βC20-H25	417 sh	447	τR_3_(A1), τR_2_(A1)
	1121	ν_a_SO_2_	409 s	445	βC14-O5
1100 w	1096	βR_1_(A1)		421	τR_2_(A1), τR_3_(A1)
1069 s	1059	νC9-C13		410	βR_2_(A2), βC13-O4
1042 vs	1049	νS1-O6			

*^a^* For atom numbering, see Figure 2. *^b^* Abbreviations: ν, stretching; β, in the plane bending; γ, out-of-the-plane bending; wag, wagging; τ, torsion; ρ, rocking; τw, twisting; δ, deformation; a, antisymmetric; s, symmetric; A1, A2, A3, and six member’s rings R1, R2, and R3, respectively; W, water. In the assignments, only contributions with calculated PEDs above 10% are indicated. In the experimental spectra, numbers are followed by a literal approximate description of the band intensity (vs, very strong; s, strong; m, medium; w, weak; sh, shoulder).

**Table 11:** Comparisons of scaled internal force constants for the anion, anhydrous, and monohydrated salts of ARS in the gas phase by using the B3LYP/6-311++G** level of theory ^a^.

Force Constants	ARS-Na	ARS-Na/H_2_O	ARS^−^
*f*(νO-H)_Water_		7.47	
*f*(νO-H)_H-bonded_	5.71	5.71	4.95
*f*(νC-H)	5.15	5.15	5.12
*f*(νC=O)	10.35	10.23	10.07
*f*(νS=O)	7.02	7.04	7.21
*f*(νS-O)	6.79	6.78	
*f*(νO^…^Na)	0.69	0.69	
*f*(δOH)_H-bonded_	1.99	1.11	1.12
*f*(δSO_2_/δSO_3_)	2.12	2.14	1.70

^a^ Units are mdyn Å^−1^ for stretching and mdyn Å rad^−2^ for angle deformations.

## Data Availability

Data is available upon request to the corresponding author.

## Supplementary Materials

The following supplementary materials can be downloaded at: https://www.mdpi.com/article/10.3390/molecules30153286/s1, Tables S1 and S2 with the results of the AIM calculations and calculated IR wavenumbers for the studied ARS species, and Figures S1–S3, with literature structural data for ARS analogues, the magnitudes and orientations of the dipole moments for the studied ARS species in gas phase, and calculated MK and NPA charges for relevant atoms in the different ARS studied species.
