# Supplementary material for: Structural and Vibrational Characterizations of Alizarin Red S
Source: Molecules. 2025 Aug 5;30(15):3286. doi: 10.3390/molecules30153286 (PMC12348708; doi:10.3390/molecules30153286)
Supplement: Supplementary file 1 [file molecules-30-03286-s001.zip › molecules-3705495-supplementary.pdf]

Structural and Vibrational Characterizations of Alizarin Red S

César A. N. Catalán,[a] Licínia L. G. Justino,[b,*] Rui Fausto,[b,c] Gulce O. Ildiz[c]

and Silvia Antonia Brandán[a,*]

[a] Cátedra de Química General, Instituto de Química Inorgánica, Facultad de Bioquímica, Química y Farmacia, Universidad Nacional de Tucumán, Ayacucho 471, (4000) San Miguel de Tucumán, Tucumán, Argentina.

[b] CQC-IMS, Department of Chemistry, University of Coimbra, Rua Larga, 3004-535 Coimbra, Portugal.

[c] Spectroscopy@IKU, Faculty of Sciences and Letters, Department of Physics, Istanbul Kultur University, Ataköy Campus, Bakirköy 34156, Istanbul, Turkey.

[*] Corresponding authors. Tel.: +54-381-4247752; fax: +54-381-4248169;

E- mail: silvia.brandan@fbqf.unt.edu.ar; brandansa@yahoo.com.ar (S. A. Brandán).

liciniaj@ci.uc.pt (L. L. G. Justino).

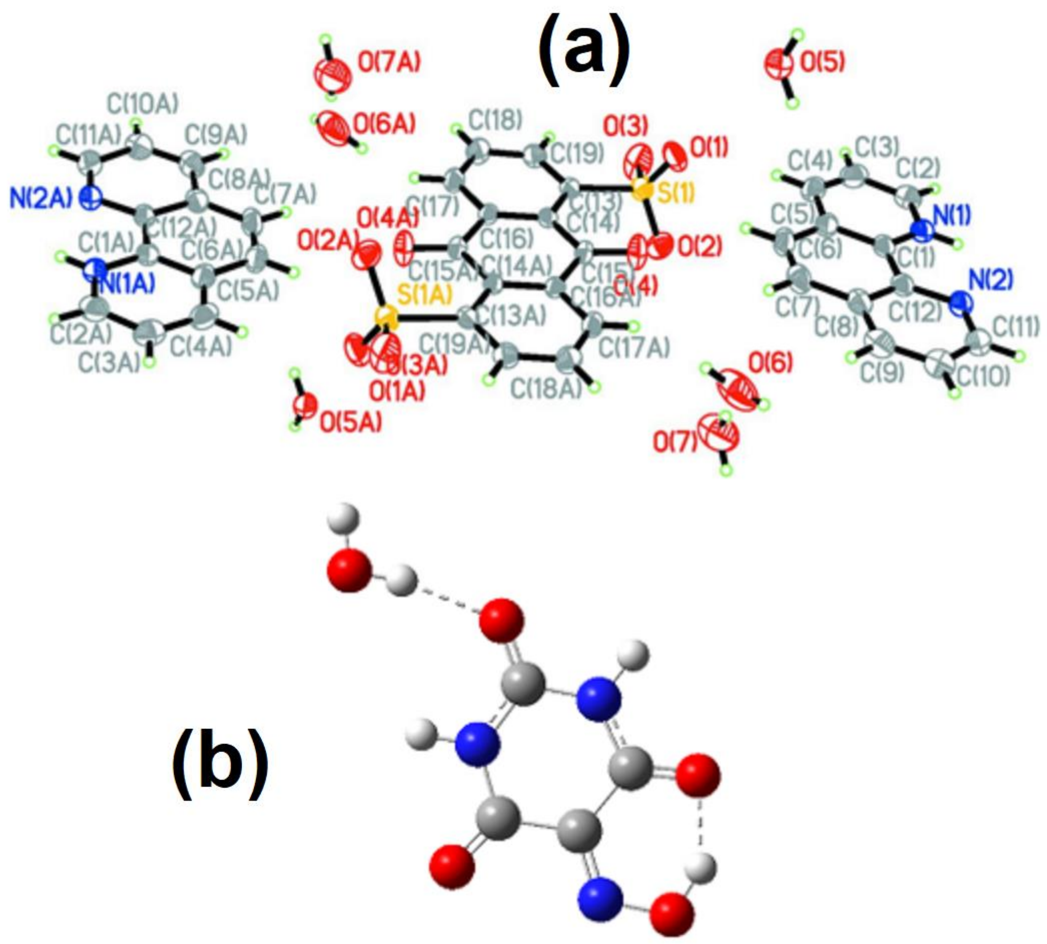

Figure S1. Structures of (a) Bis(1,10-phenanthrolin-1-ium) 9,10-dioxo-9,10-dihydroanthracene-1,5-disulfonate hexahydrate [31] and (b) monohydrate vialuric acid [27,28].

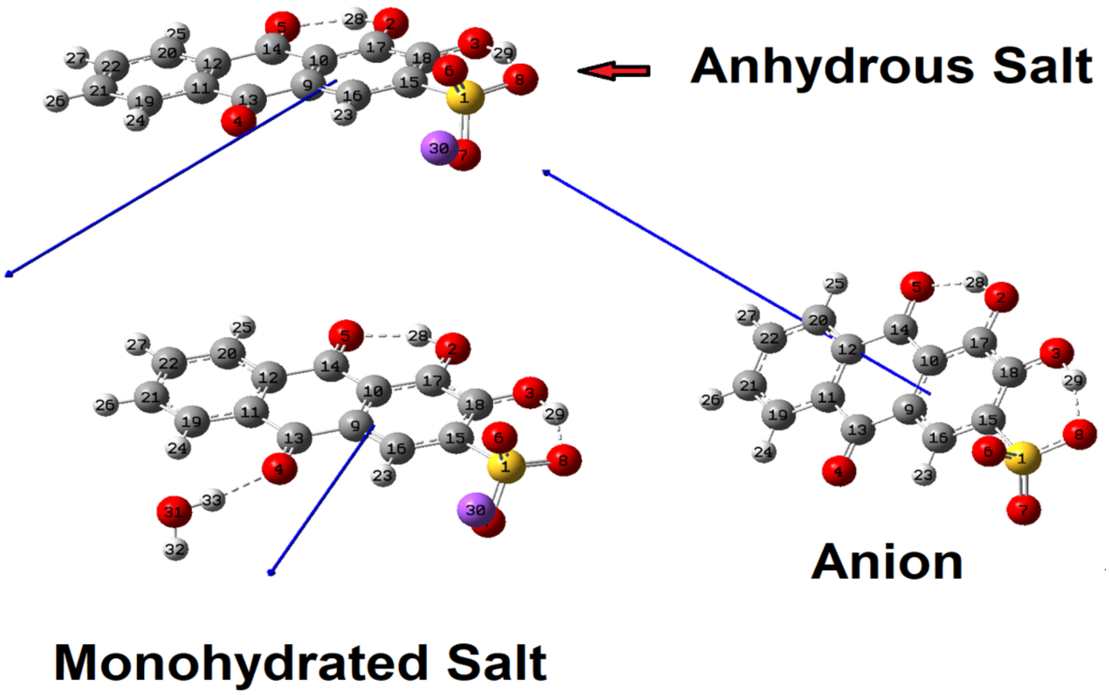

Figure S2. Magnitudes and orientations of dipole moment vectors of ARS anion, and anhydrous and monohydrated ARS Na^+ salts in gas phase, calculated at the B3LYP/6-311++G** level.

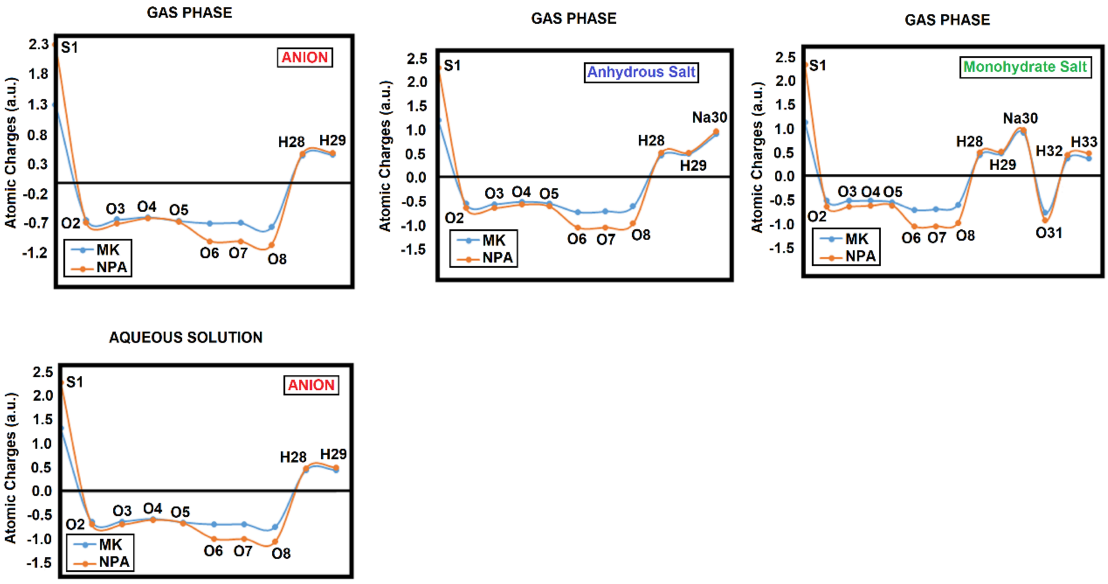

Figure S3. B3LYP/6-311++G** calculated Merz-Kollman (MK) and natural population atomic (NPA) charges on H-bond acceptor and donor groups of anion, anhydrous and monohydrated salts of ARS in gas phase and for the anion in aqueous solution.

Table S1. Analysis of the bond critical points (BCPs) and ring critical point (RCPs) for the anion, and anhydrous and monohydrated salts of ARS (B3LYP/6-311++G** data).[a]

**ANION**							
GAS PHASE							
	H28···O5	H29···O8	RCP1	RCP2	RCP3	RCPN1	RCPN2
$\rho(r)$	0.0517	0.0631	0.0219	0.0170	0.0203	0.0199	0.0184
$\nabla^2\rho(r)$	0.1492	0.1568					
Distance	1.671	1.578					
AQUEOUS SOLUTION							
	H28···O5	H29···O8	RCP1	RCP2	RCP3	RCPN1	RCPN2
$\rho(r)$	0.0507	0.0475	0.0218	0.0170	0.0207	0.0195	0.0167
$\nabla^2\rho(r)$	0.1484	0.1372					
Distance	1.678	1.697					
ANHYDROUS SALT							
GAS PHASE							
	H28···O5	H29···O8	RCP1	RCP2	RCP3		
$\rho(r)$	0.0501	0.0450	0.0219	0.0169	0.0203		
$\nabla^2\rho(r)$	0.1456	0.1420					
Distance	1.683	1.708					
	O6···Na	O7···Na	RCPN1	RCPN2	RCPN3		
$\rho(r)$	0.0265	0.0263	0.0196	0.0166	0.0183		
$\nabla^2\rho(r)$	0.1666	0.1650					
Distance	2.244	2.247					
MONOHYDRATED SALT							
GAS PHASE							
	H28···O5	H29···O8	RCP1	RCP2	RCP3		
$\rho(r)$	0.0501	0.0449	0.0214	0.0166	0.0198		
$\nabla^2\rho(r)$	0.1460	0.1414					
Distance	1.684	1.710					
	O6···Na	O7···Na	RCPN1	RCPN2	RCPN3	RCPN4	
$\rho(r)$	0.0271	0.0265	0.0196	0.0166	0.0184	0.0085	
$\nabla^2\rho(r)$	0.1738	0.1684					
Distance	2.233	2.243					
	CH24···O31	OH33···O4					
	0.0123	0.0248					
	0.0376	0.0951					
	2.362	1.926					

[a] Electron densities, ρ(r) in e bohr^{-3}; $\nabla^2\rho(r)$ in e bohr^{-5}; r in bohr (e = 1.602 × 10^{-19} C; 1 bohr = 0.529 Å); distances in Å.

Table S2. Calculated (SQMFF, B3LYP/6-311++G**) wavenumbers (cm^{-1}) and assignments for the isolated ARS^{-}, ARS-Na and ARS-Na/H_2O species.[a]

ARS-Na		ARS-NA/H_2O		ARS^{-}	
ν	Assignments[b]	ν	Assignments[b]	ν	Assignments[b]
		3730	ν_aOH(W)		
		3519	ν_sOH(W)		
3241	νO3-H29	3240	νO3-H29	3173	νO2-H28
3177	νO2-H28	3175	νO2-H28	3072	νC16-H23
3079	νC16-H23	3082	νC16-H23	3067	νC20-H25
3072	νC20-H25	3072	νC20-H25	3064	νC19-H24
3069	νC19-H24	3069	νC19-H24	3043	νC22-H27
3053	νC22-H27	3055	νC21-H26	3027	νC21-H26
3039	νC21-H26	3041	νC22-H27	2811	νO3-H29
1650	νC13=O4	1636	νC13=O4	1641	νC13=O4
1618	νC14=O5	1623	νC14=O5	1605	νC14=O5
1575	νC20-C22	1573	νC9-C16	1575	νC11-C19
1565	νC9-C16	1571	νC19-C21	1554	νC11-C12
1552	νC11-C12	1550	νC21-C22,νC14=O5	1541	νC9-C16,νC14=O5
		1546	δOH(W)		
		1546	δOH(W)		
1524	νC15-C18,νC10-C17	1471	δO3-H29,νC9-C10	1520	δO2-H28,νC15-C18
1460	βC20-H25,βC19-H24	1461	δO3-H29,βC19-H24	1458	βC19-H24
1451	νC9-C10,βC16-H23	1460	βC16-H23	1454	δO3-H29
1438	βC22-H27	1437	βC22-H27,βC21-H26	1447	νC9-C10
1432	νC10-C17	1421	δO3-H29,νC15-C18	1434	βC21-H26,βC22-H27
1391	δO3-H29	1351	νC10-C14,νC10-C17, δO2-H28	1407	νC10-C17
1332	νC10-C14	1327	νC17-O2,βR_1(A3)	1340	νC10-C14, δO2-H28
1321	νC17-O2,δO2-H28	1312	νC11-C19, νC12-C20,νC11-C12	1308	νC17-O2
1309	νC11-C19,νC12-C20	1296	νC9-C13	1303	νC12-C20
1284	νC9-C13,νC11-C13	1265	βC19-H24	1299	νC15-C18
1256	βR_1(A2),βC20-H25	1255	νC16-C15,νC18-O3	1268	νC16-C15,νC18-O3
1248	νC16-C15,νC18-O3	1225	νC12-C14	1251	βC20-H25
1216	νC12-C14	1189	νC11-C13	1219	βC16-H23,νC12-C14
1185	νC11-C13	1167	βC16-H23	1180	νC11-C13
1166	βC16-H23	1156	βC21-H26,βC22-H27,βC20-H25	1171	$\nu_a SO_3$
1149	βC22-H27,βC21-H26	1121	$\nu_a SO_2$	1163	βC16-H23
1119	$\nu_a SO_2$	1096	βR_1(A1)	1142	βC22-H27,βC21-H26
1089	βR_1(A1)	1059	νC9-C13	1085	βR_1(A1)
1054	νC9-C13	1049	νS1-O6	1079	$\nu_a SO_3$
1045	νS1-O6	1044	νC21-C22	1060	νC9-C13
1040	βC14-O5	1019	γC21-H26,γC19-H24	1037	βC14-O5
1009	γC21-H26,γC22-H27	1011	νC21-C22,νC20-C22	1004	νC21-C22,νC19-C21
1007	νC21-C22νC19-C21	1000	γC20-H25,γC22-H27	997	γC21-H26
992	γC20-H25,γC19-H24	944	$\nu_s SO_2$	984	γC20-H25
943	$\nu_s SO_2$	913	γC20-H25,γC19-H24	925	$\nu_s SO_3$
905	γC20-H25,γC19-H24	905	βC13-O4	919	γC16-H23
904	βC13-O4	898	γC16-H23	901	βC13-O4
888	γC16-H23	854	νC17-C18	896	γC19-H24
854	βR_2(A3),βC14-O5	801	γC22-H27,γC21-H26	851	νC17-C18
799	γC22-H27, γC21-H26	771	βR_1(A2)	829	τwO3-H29
772	γC14-O5,γC13-O4	769	τR_1(A1), τR_1(A2), γC13-O4	793	γC22-H27
768	βR_1(A2), βR_1(A3)	740	τR_2(A3),γC17-O2,γC18-O3	769	βR_1(A3),βR_1(A2)
735	γC17-O2,γC18-O3	729	βR_2(A1)	762	γC14-O5,γC13-O4
728	βR_2(A1)	726	τR_1(A3),γC18-O3	728	βR_2(A1), βR_3(A1)
723	τR_1(A3),γC18-O3	705	τwO2-H28	727	γC18-O3
701	τwO2-H28	682	τwO3-H29	719	γC18-O3,τR_1(A3)
673	βR_3(A1)	673	βR_3(A1)	701	τwO2-H28
671	τR_1(A1)	671	τR_1(A1), τR_2(A1)	671	βR_3(A1),βR_2(A1)
661	τwO3-H29	655	βR_2(A3), βC18-O3	666	τR_1(A1)
653	βR_2(A3), βC18-O3	630	wagSO_2	653	βC18-O3
631	wagSO_2	590	τR_3(A3),γC15-S1	629	$\delta_s SO_3$
590	τR_3(A3), γC15-S1	587	τR_3(A3),γC15-S1	586	τR_3(A3),γC17-O2
584	βR_3(A1),τR_3(A3)	561	τH33-O4,τO31-H33	582	$\delta_s SO_3$,βR_3(A1)
548	τR_1(A3)	550	τR_1(A3)	540	τR_1(A3)

531	τwSO2,δSO2	527	τwSO$_2$, δSO$_2$	517	δ$_a$SO$_3$
508	βR$_3$(A3), βR$_2$(A1)	510	βR$_3$(A3),βR$_2$(A1)	499	δ$_a$SO$_3$,βR$_3$(A3)
476	δSO$_2$,τwSO$_2$	478	δSO$_2$, τwSO$_2$	485	δ$_a$SO$_3$
469	βR$_3$(A2),βR$_3$(A3)	472	βR$_3$(A2), βR$_3$(A3)	468	βR$_3$(A2),βR$_3$(A3)
447	τR$_3$(A1),τR$_2$(A1)	447	τR$_3$(A1),τR$_2$(A1)	445	βC14-O5
445	βC14-O5	445	βC14-O5	445	τR$_3$(A1)τR$_2$(A1)
420	τR$_2$(A1), τR$_3$(A1)	421	τR$_2$(A1), τR$_3$(A1)	418	τR$_2$(A1) τR$_3$(A1)
405	βC17-O2	410	βR$_2$(A2), βC13-O4	406	βC17-O2
377	δO6S1C15	381	δO6S1C15	379	βC18-O3
373	βC18-O3	373	βC18-O3,βC17-O2	372	γC18-O3,βC18-O3
367	ρSO$_2$	367	ρSO$_2$	355	ρSO$_3$
324	νO6-Na30,βC17-O2	327	νO6-Na30	317	βR$_2$(A2)
312	βR$_2$(A2)	315	βR$_2$(A2)	290	ρSO$_3$
287	τR$_3$(A3)	291	τR$_3$(A3)	288	ρSO$_3$
264	ρSO$_2$	275	τH33-O4	262	βC15-S1
247	νO6-Na30	262	ρSO$_2$,τwSO$_2$	224	ρ'SO3,ButC10-C11
238	δO6-Na30	251	νO6-Na30	203	βR$_2$(A3)
204	δO6-Na30,νO6-Na30	241	δO6-Na30	170	ButC6-C8
196	βR$_2$(A3),νC15-S1	206	νO6-Na30,δO6-Na30	137	τR$_2$(A3)
168	ButC6-C8, ButC10-C11	200	βR$_2$(A3),νC15-S1	123	βC15-S1
138	τR$_2$(A3)	168	ButC6-C8	114	τR$_1$(A2)
128	βC15-S1	148	νO4-H33,βC15-S1	102	γC15-S1
116	τR$_1$(A2)	140	τO31-H33,τR$_2$(A3)	66	τR$_2$(A2)
100	γC15-S1	136	τO31-H33	31	τR$_3$(A2)
66	τR$_2$(A2)	113	νO4-H33,τR$_1$(A2)	24	τwSO$_3$
41	τO6-Na30	113	νC13=O4,τH33-O4		
33	τR$_3$(A2)	101	γC15-S1,τR$_3$(A2)		
11	τS1-C15,τwO3-H29	67	τR$_2$(A2)		
		55	δH33O4C13,δO31H33O4		
		40	τO6-Na30		
		34	τwO4-C13,τR$_2$(A2)		
		24	τwO4-C13		
		17	τS1-C15		

[a] For atom numbering see Fig. 2. [b] Abbreviations: ν, stretching; β, in the plane bending; γ, out of the plane bending; wag, wagging; τ, torsion; ρ, rocking; τw, twisting; δ, deformation; a, antisymmetric; s, symmetric; A1, A2, A3, six member's rings R1, R2 and R3, respectively; W, water. In the assignments, only contributions with calculated PEDs above 10% are indicated.
